# Deep Generative Models-Based Anomaly Detection for Spacecraft Control Systems

**DOI:** 10.3390/s20071991

**Published:** 2020-04-02

**Authors:** Hyojung Ahn, Dawoon Jung, Han-Lim Choi

**Affiliations:** 1 Korea Aerospace Research Institute, Daejeon 34133, Korea; hjahn@kari.re.kr (H.A.); dwjung@kari.re.kr (D.J.); 2 Department of Aerospace Engineering, Korea Advanced Institute of Science and Technology, Daejeon 341141, Korea

**Keywords:** anomaly detection, spacecraft, attitude control system, deep-learning, generative model

## Abstract

A spacecraft attitude control system provides mechanical and electrical control to achieve the required functions under various mission scenarios. Although generally designed to be highly reliable, mission failure can occur if anomalies occur and the attitude control system fails to properly orient and stabilize the spacecraft. Because accessing spacecraft to directly repair such problems is usually infeasible, developing a continuous condition monitoring model is necessary to detect anomalies and respond accordingly. In this study, a method for detecting anomalies and characterizing failures for spacecraft attitude control systems is proposed. Herein, features are extracted from multidimensional time-series data of a simulation of the attitude control system. Then, the artificial neural network learning algorithms based on two types of generation models are applied. A Bayesian optimization algorithm with a Gaussian process is used to optimize the hyperparameters for the neural network to improve the performance. The performance is evaluated based on the reconstruction error through the algorithm using the newly generated data not used for learning as input data. Results show that the detection performance depends on the operating characteristics of each submode in the operation scenarios and type of generation model. The diagnostic results are monitored to detect anomalies in operation modes and scenarios.

## 1. Introduction

Complex systems can sometimes fail or behave abnormally, which can affect the success or failure of a given mission. Therefore, detection of these related phenomena occurring in the system is important for decision making in order to successfully perform a mission. Several statistical methods have been developed to address various issues including, more recently, machine learning techniques [1]. The two main challenges associated with failure detection are fault diagnosis (FD) and anomaly detection (AD): FD is intended to determine what failure occurred, and AD is used to determine the extent of the deviation from the proper operating range of the system due to the failure [2]. AD does not rely on prior information about the type or form of failure as it only determines if the system is operating outside of its normal range. This approach is gaining attention as a way to evaluate the health of large, complex systems, such as aerospace systems. However, it is difficult to know in advance the mechanism by which faults occur and to obtain data regarding fault conditions because most aerospace systems are designed with high-reliability [3,4]. Further, in complex systems, anomalies occur via complicated pathways. Therefore, a method for processing multidimensional data coming from multiple channels is required.

In this study, a spacecraft system is considered as a representative example of a typical large system in aerospace. A spacecraft system anomaly is defined as any mission-degrading event affecting an on-orbit operational spacecraft. Examples include onboard computer errors or failures, attitude control system malfunctions, radio contact loss, solar panel efficiency degradation, and many other mechanical and electronic symptoms [5]. In particular, three anomalies can have catastrophic consequences for the entire spacecraft system: loss of control related to sensor failure, input voltage drop, and abnormal temperature rise of boards or sensors [5]. A functioning spacecraft attitude control system detects attitude errors caused by external disturbances and uses them to stabilize the spacecraft’s position and orbit. These disturbances can include gravity differences owing to the spacecraft altitude, magnetic field interactions, altitude-related solar radiative pressure changes, or movement in antennas or solar panels [6]. Using various sensors such as gyroscopes, star sensors, solar sensors, and earth sensors, the control system stabilizes the spacecraft in the desired direction by rotating the spacecraft itself or its own mass within the spacecraft. If a failure occurs within the system and the system fails to perform the required function of orienting and stabilizing the spacecraft, the spacecraft can become inoperable. Due to the limited physical accessibility of spacecraft, these problems can be impossible to directly repair. Therefore, it is necessary to respond through continuous condition monitoring, and AD is required to improve autonomy and stability in the operation of spacecraft missions.

Time-series data transmitted from each subsystem, part, and sensor are needed to diagnose the spacecraft system’s mechanical condition. Data analysis methods and statistical techniques for detecting anomalies using time-series data have long been studied using traditional data-processing techniques [7,8,9,10]. Some studies utilize such techniques in a way that reflects the characteristics of the data used. For example, one study has proposed an integrated model of the mixture of probabilistic principal components analyzers (MPPCA) [11] for housekeeping data, in particular the real-valued continuous variables and categorical mixture distribution for modeling categorical discrete variables [12]. However, in the data feature extraction step, existing traditional data-processing techniques require specialized knowledge of related areas, costing much effort and time to process the data. In addition to the emergence of deep-learning techniques, new models for time-series analysis and prediction have been developed, reducing the need for specialists in the feature extraction stage. Under these models, data features can be extracted by learning through algorithms; the deep-learning method allows data features to be extracted through deep-learning algorithms. Recently, numerous methods and combinations thereof have been applied, e.g., convolutional neural network, recurrent neural network, long short term memory (LSTM), autoencoder (AE), generative adversarial network (GAN), and variational autoencoder (VAE).

Some researches reviewed major deep-learning techniques that can be used to diagnose abnormalities in time-series data and demonstrated the effectiveness and advantages of deep-learning applications over existing statistical techniques by presenting high-performance research examples [13,14,15,16]. Feature extraction learning models of multivariate time-series data have been presented using the deep-learning technique [14], but the networks used were not deep, and the size of the data set was not particularly large [15]. O’Meara presented three applications of deep-learning neural networks for analyzing spacecraft telemetry data: an AE neural network for feature vector extraction and AD, and a multi-layer LSTM network for telemetry forecasting and anomaly prediction [16]. The general conclusion reached after initial experiments and iterative algorithm changes was that neural networks are powerful tools for many interesting applications in spacecraft telemetry monitoring. In the automated telemetry health monitoring system developed in their paper, neural networks were ultimately created: AE neural network for feature vector extraction and AD and multi-layer LSTM network for remote measurement and anomaly predictions [16,17,18,19,20]. The previous research results show that deep-learning offers key advantages over existing statistical techniques of feature extraction for spacecraft systems, in which there are large amounts of data to be processed.

In this study, methods for detecting anomalies in a spacecraft attitude control system using deep learning technology are evaluated. This study aims to verify that the proposed AD method based on the selected neural network model is suitable for diagnosing the state of the spacecraft attitude control system. Measuring AD performance requires a healthy differential model, so we use simulation data that can be clearly labeled as normal and abnormal. The data set utilizes soft-sensor-based data generated by LUNar Attitude and Orbit Control System (AOCS) SIMulator (LUNASIM) [21], a satellite attitude control simulator developed in-house at the Korea Aerospace Research Institute (KARI) to test the performance of attitude control flight software for the Korea Pathfinder Lunar Orbiter (KPLO) [22]. A series of processes for AD using multidimensional time series spacecraft AOCS data includes the following works: (a) preprocessing of multi-channel time series data (including purification, integration, cleanup and transformation) and data feature extraction using deep neural networks; (b) selecting diagnostic methods based on generative models considering actual data conditions (e.g. number of samples and level of labeling); (c) improving algorithm performance and robustness by applying automatic determination of hyperparameters using Bayesian optimization (BO) with a Gaussian process (GP) and data processing considering the characteristics of physical model and applied deep networks; (d) reviewing the suitability and validity of the system by comparing the predicted results between two selected generation models (VAE [23] and generative adversarial networks AD (GANomaly) [24]); (e) presenting AD monitoring cases in the spacecraft attitude control system based on the diagnosis results.

## 2. Problem and Data Description

This section describes the problem to be solved, the simulator used to generate data and characteristics of the data.

### 2.1. Problem Description

A spacecraft attitude control system determines and controls a spacecraft’s attitude and momentum using various sensors and actuators within the limits defined by the payload pointing and the stability requirements until the mission is completed. Although the spacecraft is designed to be highly reliable in general, anomalies can lead to deviations from the requirements. Certain anomalies are apparent from the telemetry alarms, such as when a sensor fails; however, other anomalies may manifest as a collection of factors, trends, or nonlinear differences from the normal telemetry that are not apparent. Such a spectrum of anomalies presents special challenges when the spacecraft has difficulty making ground contact because of low communication bit rates, large distances, irregular contact times, and inconveniently located ground stations, such as during a lunar mission. An AD system using machine learning could be an effective tool in this case because it could proactively and semi-autonomously detect possible anomalies. In this study, we develop a model for detecting anomalies in the attitude control system signals for the development of KPLO. Table 1 shows the typical spacecraft attitude control system units and their corresponding output signals that are used by KPLO.

KPLO is KARI’s first lunar mission; therefore, the operational data is not available. We used simulated attitude control performance data generated by LUNASIM [21]. Certain aspects of the KPLO mission are similar to low-Earth and geosynchronous orbit missions; however, key differences exist that prevent us from blindly applying existing Earth-orbit AD standards. For example, a coordinate system transition occurs when entering the lunar orbit [22]. Moreover, the spacecraft experiences large angular rates and disturbances while preparing for the lunar orbit and during the lunar orbit insertion at equinoxes for reorienting the solar arrays. Large angular rates are also experienced at the apogee and perigee during the lunar transfer orbit. An AD must consider these unique data characteristics to prevent false positives. In the next section, we provide a general description of LUNASIM, its output data, and the various types of simulated faults.

### 2.2. LUNASIM

LUNASIM [21] is being developed in-house at KARI to test the performance of attitude control flight software for the KPLO, a lunar orbiter mission currently in its critical design phase. LUNASIM is composed of attitude control flight software (FSW), equipment, environment, and dynamics models, I/O handlers, and simulation infrastructure. It can run flight software in a software-only model-in-the-loop mode on a PC, or in hardware-in-the-loop mode with flight software running on an actual onboard computer (OBC) interfacing with equipment hardware and model software for environment and dynamics.

KPLO attitude control FSW switches to discrete submodes when operating. Attitude control submodes group together similar attitude control methods and parameters into a set of predictable states or control laws. Each submode behaves differently, and each is assigned different performance envelopes. In particular, some submodes may use thrusters and Sun sensors for attitude control while others do not, thus training must be done on each mode separately. Table 2 lists the submodes.

LUNASIM reads test scenarios (Table 3) as a series of pairs of event commands and times that may include submode transitions but also other maneuver commands. For example, the scenarios in Table 3 including LAM submode transitions also perform 180 degree rotation. Test scenarios may be of various durations. All scenarios start at 0, and brackets indicate the moment in time at which the mode transition occurs. For example, the first TP in LAM1 lasts from 199 to 698 s. The first mode transition usually does not start at 0 and this is because some extra (dummy) time is allocated at the beginning of scenarios to allow time for turning on equipment, etc. Scenario results are written as time-series tables in a database. All data are output at 8 Hz, matching the frequency at which flight software runs on the OBC.

Crucially, LUNASIM contains fault injection features that allow the test operator to inject a pre-defined set of faults at certain points during a simulation run. Available faults include: 1) failure of one of four reaction wheel assemblies (RWA), 2) wheel speed anomaly in RWA #1, 3) spike in gyro reference assembly (GRA)-measured spacecraft angular rate, 4) spike in course Sun sensor array (CSSA) #1 and #4. These injectable faults are adapted from a subset of fault management test code that has been developed for other spacecraft and are representative of faults that have a realistic chance of occurring on orbit.

Wheel speed anomalies and GRA and CSSA spikes can be simulated at arbitrary and multiple points during a run. We define special event commands that invoke (and cancel) these faults at the appropriate times, e.g., by setting a variable to a hard-coded ‘failure’ value. This, in turn, causes the simulator dynamics to react in a nonlinear fashion that is visible in telemetry variables but not necessarily describable in a deterministic way. Wheel failure is a special case in that it is simulate d for the entire length of a run. This is because the operation concepts are: (1) Reboot to thruster-based safe-hold mode on a wheel failure, (2) Via ground command, start using the remaining three wheels only. As we are interested in attitude control performance after exiting safe-hold mode, our wheel-failure scenarios assume that the failure has already occurred and continues indefinitely.

LUNASIM also models equipment noise such as gyro rate and angular noise over time, sun sensor current fluctuations, wheel voltage noise, etc. While a full treatment of LUNASIM noise models is beyond the scope of this work, the faults and maneuvers triggered in the test scenarios typically result in instantaneous signals with magnitudes that are many times greater than the mean and are visually distinguishable from noise, and with diminishing but persistent magnitudes afterward.

### 2.3. Data Schema

For every simulation run, LUNASIM writes a total of 31 tables, grouped roughly by functionality. These tables include spacecraft rate and stability, control error, knowledge error, various equipment (e.g., star trackers, thrusters, Sun sensors, solar array drive mechanisms (SADMs), RWAs, and GRAs), Kalman filter, disturbance torques, and current attitude control submode. While each table contains a different number of columns, each table follows the same general layout: a time column as the primary key (simulation time, starting from 0 s), followed by columns that usually map to variables used in simulator models or flight software. Such variables may represent continuous or discrete (e.g., true/false) variables. A sample table schema is shown in Table 4.

For this work, we concatenated (left join) all tables on their time columns and regrouped data by attitude control submode, creating a table for each submode (LAM, SP, TSH, TP, WOL; see Table 2). Attitude control submodes group together similar attitude control methods and parameters into a set of predictable states or control laws. Each submode behaves differently, and each is assigned different performance envelopes, allowing training on each mode separately. In particular, some submodes may use thrusters and Sun sensors for attitude control while others do not. For this reason, the set of injectable failures also differs based on the submode.

Table 5 gives a summary of faults injected per scenario.

## 3. Preliminaries

In this study, the model learns using only normal data in an unsupervised mode, and then it is applied to abnormal data as an AD model. This method then detects anomalies by measuring the reconstruction error when new data is input. The key is that restoration would not work properly for abnormal data, because the generative model was trained using only normal data. This, in turn, means that the reconstruction error of a given sample is a good estimator for detecting an abnormal state. In this study, we utilize two unsupervised learning algorithms to detect abnormal states solely based on normal data sets: VAE [23] and GANomaly [24].

### 3.1. Variational Autoencoder (VAE)

VAE is an improvement on existing autoencoders (AE) [25], and it differs in that it samples latent code from a probability distribution. An AE always generates the same code for the same input, but VAE is a probabilistic model that tries to reconstruct data from the input data space via maximum likelihood estimation and variational inference. The model comprises two parts, encoder and decoder. The key is that VAE trained with only normal data cannot reconstruct abnormal data well; this means the reconstruction error of a given sample is a good estimator for detection of an abnormal state. In VAE, the mean and standard deviation of a normal distribution are estimated through the encoder, and the data *x* are restored by sampling the code *z,* ($e_{\phi }:x\to z)$. Further, we could generate new samples similar to the original input data (*x*) by inputting the codes sampled from the distribution *p*(*z*) of the latent vectors (*z*) to the decoder, ($d_{\theta }:z\to x)$. [26]. In VAE, the encoder and decoder model parameters of the conditional distributions are denoted as $q_{\phi }\left(z|x\right)$ and $P_{\theta }\left(x|z\right)$, which were assumed to be Gaussian distributions. The cost function for training the model is as follows:

(1) $$L_{\upsilon }\left(x,\;\phi ,\;\theta \right)=E_{q\left(z|x\right)}\left[∥x-d_{\theta }\left(z\right){∥}_{2}^{2}\right]+\lambda D_{KL}\left(q_{\phi }\left(z|x\right)∥p\left(z\right)\right),$$

where *θ* and *ϕ* denote hidden parameters of neural nets and $\lambda =\sigma_{x}^{2}$ is a tuning parameter equal to a known variance of data [26]. $E_{q\left(z|x\right)}\left[∥x-d_{\theta }\left(z\right){∥}_{2}^{2}\right]$ is the reconstruction error and upon minimizing it through backpropagation, the AE is trained. $D_{KL}\left(q_{\phi }\left(z|x\right)∥p\left(z\right)\right)$, the Kullback–Leibler divergence [27], is aimed to minimize the reconstruction loss of x during training. An important part of the training is the reparameterization trick, which creates random decoder inputs in the following manner: $z=\mu_{z}+\sigma_{z}\epsilon$, where $\mu_{z}$ and $\sigma_{z}$ are outputs of the encoder, and ϵ is the sampled form p(z) [26].

### 3.2. GANomaly

GANs are holistic models for reconstructing data from a latent space by optimizing Jensen Shannon Divergence (JSD) of generated and real-world samples [28]. The first GAN-based attempt to detect anomalies was AnoGAN [29]. AnoGAN trains on a GAN in which the generator attempts to deceive the discriminator, while the discriminator attempts to discriminate the authenticity of a given normal datum. This kind of competitive play can sample normal data from latent variables of a probability distribution function. However, AnoGAN algorithm is not automated end-to-end because it needs an additional optimization process. In our work, we adopted a popular GAN-based AD framework: GANomaly. GANomaly borrows an AE-style generator to solve the additional optimization problem, and an encoder-style discriminator for parsing the representation of a given training data set. Overall, the objective function for the generator is as follows:

(2) $$L=w_{adv}L_{adv}+w_{con}L_{con}+w_{enc}L_{enc}\;,$$

where $w_{adv}$, $w_{con}$, and $w_{enc}$ are the weighting parameters that adjust the impact of the individual losses to the overall objective function [24]. Adversarial loss ($L_{adv}$) is the difference between the output from discriminator (*f(x)*) and output from autoencoder (*f(*$\hat{x}$*)*) to reduce the instability of GAN training [24]. Contextual loss ($L_{con}$) is to optimize toward the learning contextual information about the input data (*x*) by measuring the distance between the input (*x*) and the generated images ($\hat{x}$) [24]. Encoder loss ($L_{enc}$) is to minimize the distance between the bottleneck features of the input (*z*) and the encoded features of the generated image ($\hat{z}$) [24].

### 3.3. Bayesian Optimization

BO typically works for continuous functions by assuming the unknown function sampled from a GP and maintaining a posterior distribution for this function as observations are made or as the results of running learning algorithm experiments with different hyperparameters are observed [30]. This method can be used for carefully tuning the learning parameters and model hyperparameters in machine learning algorithms. Snoek et al. [30] presented methods and improved results for performing BO for hyperparameter selection of general machine learning algorithms.

We determine the optimized parameter:

(3) $$p^{**}=argmax_{p\in A}f\left(p\right),$$

where, *A* is the space of the possible hyperparameters, and f is the objective function. We assumed that *f(p)* is a black-box function that does not know what the output is when the input is inserted. Based on the observed data $D_{i}=\left[\left(p_{1},\;f\left(p_{1}\right)\right),\;\left(p_{2},\;f\left(p_{2}\right)\right)\cdots \left(p_{n},\;f\left(p_{n}\right)\right)\right],$ the function *f(p)* is estimated using the GP prior:

(4) $$f\left(p\right)\sim GP\left[m\left(p\right),\;k\left(p,\;p^{\prime }\right)\right].$$

The BO principle is based on the following Bayes’s theorem:

(5) $$P\left(f|D_{i}\right)\propto \;P\left(D_{i}|f\right)*P\left(f\right),$$

where $P\left(f|D_{i}\right)$ is the posterior probability of a model knowing the data; $P\left(D_{i}|f\right)$ is the likelihood of the data $D_{i}$ knowing model f; and P(f) is the prior probability of f. The function $f\left(p_{i}\right)$ is moved to the next observation point $\;\left(p_{n+1},\;f\left(p_{n+1}\right)\right)$ with the acquisition function. We use the Matérn kernel, which is a stationary kernel and a generalization of the RBF kernel [31]. It has an additional parameter ν that controls the smoothness of the resulting function. It is parameterized by a length-scale parameter l > 0, which can either be a scalar (isotropic variant of the kernel) or a vector with the same number of dimensions as the inputs x (i.e., anisotropic variant of the kernel). The kernel is given by:

(6) $$k\left(p_{i},p_{j}\right)=\sigma^{2}\frac{1}{\Gamma \left(\nu \right)2^{\nu -1}}{\left(\gamma \sqrt{2\nu }d\left(p_{i}/l,\;p_{j}/l\right)\right)}^{\nu }K_{\nu }\left(\gamma \sqrt{2\nu }d\left(p_{i}/l,\;p_{j}/l\right)\right).$$

As ν→∞, the Matérn kernel converges to the RBF kernel. When ν = 1/2, the Matérn kernel becomes identical to the absolute exponential kernel. In particular, ν = 3/2 and ν = 5/2 are popular choices for learning functions that are not infinitely differentiable (as assumed by the RBF kernel) but are at least once (ν = 3/2) or twice differentiable (ν = 5/2).

The acquisition function uses the most commonly used expected improvement (EI) function. Based on the objective function estimated to date, the EI outputs a number indicating the availability of the input value (*p*) by considering the probability improvement of deriving a function value greater than the maximum function value (*f(p^+^) = max_i_ f(p_i_)*) of the points (*f(p*_1_*), f(p*_2_*),…, f(p_n_)*) investigated so far and the difference between the function value and *f(p^+^)*. The expression of EI when using GP is summarized as follows:

(7) $$p^{+}=argmax_{p_{n}}f\left(p_{n}\right),$$

(8) $$\begin{array}{l} \mathrm{EI}\left(p\right)=E\left[max\left(f\left(p\right)-f\left(p^{+}\right),\;0\right)\right]\\ =\{\begin{array}{c} \left(\mu \left(p\right)-f\left(p^{+}\right)-\xi \right)\Phi \left(\frac{\mu \left(p\right)-f\left(p^{+}\right)-\xi }{\sigma \left(p\right)}\right)+\sigma \left(p\right)\phi \left(Z\frac{\mu \left(p\right)-f\left(p^{+}\right)-\xi }{\sigma \left(p\right)}\right),\;\;if\;\sigma \left(p\right)>0\\ 0,\;\;if\;\sigma \left(p\right)=0\; \end{array} \end{array},$$

where ξ is a parameter that controls the relative intensity between the exploration and exploitation; ϕ is the normal probability density function; and Φ is the cumulative density function.

## 4. Method

As most spacecraft operate in a normal state, far fewer samples of abnormal data can be acquired than those of normal data. To resolve this problem, a probability-based unsupervised AD model is trained. After training the generation model using only normal data, abnormal data can be distinguished from normal data by measuring the reconstruction error when new simulated data not used for learning are input. This study aims to validate that the proposed AD method based on the selected generation model is suitable for diagnosing the status of a spacecraft attitude control system. The spacecraft attitude control system is operated in various scenarios according to the range of missions it was intended for, and each scenario included several operation modes. To measure AD performance of the two generation models considered in this study according to the data characteristics acquired for each submode operated in the operating scenarios, a healthy probabilistic model for discrimination is required. For this reason, the simulation data that can be clearly labeled with normal and abnormal status are used. The data sets are generated using LUNar Attitude and Orbit Control System (AOCS) SIMulator (LUNASIM) [21], a spacecraft attitude control simulator developed in-house at the Korea Aerospace Research Institute (KARI) to test the performance of attitude control flight software for the Korea Pathfinder Lunar Orbiter (KPLO) [22]. The training data for the learning model comprises normal data, and the test data comprises normal and anomaly data.

Furthermore, a series of processes for AD using multidimensional time-series spacecraft AOCS data include data preprocessing (including purification, integration, cleanup, and transformation), feature extraction using deep networks, and learning models represented by algorithms considering robustness and performance optimization. Additionally, a selection method based on a generation model considering the actual data status (i.e., the sample count and labeling level) and reviews of conformity and applicability are evaluated by comparing the prediction results between two selected generation models (VAE and GANomaly). Finally, cases of AD monitoring in a spacecraft attitude control system based on the diagnostic results are summarized.

### 4.1. Data Preprocessing

Each data file is a time-series sampled at 8 Hz, with 1063 values (i.e., columns and variables) for each timestamp. The range of each variable is substantially different; therefore, a normalization process is required. Without a normalization process, a learning model can be created that relies only on specific variables over a very large range [32].

We use min-max normalization because it is more stable than standardization such that all normalized data fall between 0 and 1, as shown in the following equation:

X = (x − x_min_)/(x_max_ − x_min_), (9)

where x_min_ is the minimum value encountered for a given variable over the entire data set, and x_max_ is the maximum value encountered for a given variable over the entire data set. However, the min–max normalization may be inaccurate in the presence of outliers. The data set used in this paper did not have any outliers in the simulated data; therefore, we chose the min–max normalization method.

### 4.2. Algorithms

In this paper, the data set generated from LUNASIM is labeled with information about normal and abnormal. In most real systems, the normal state data are much larger than the abnormal state. Considering the data status, we select the AD model using only the normal data for training. Then, we predict the state of the newly input data, which include normal and abnormal data acquired by LUNASIM. Considering the large number of datasets in the system, we use the convolutional neural network (CNN)-based approach to maximize the class by extracting the “optimized” features directly from the raw data for the problem.

In this paper, we use the AD methods based on generation models, which include GANomaly presented as the latest AD method based on GAN and AE-based VAE. In the problem of image restoration based on the existing AE and GAN, 2-D convolution and 2-D transposed convolution are mostly used for the encoder and decoder, respectively. CNN is specially modeled and produced for 2D signals; therefore, the appropriate conversion from 1D to 2D is made for application to 1D signal processing, such as time-series data [33].

However, the 2D-CNN technique suffers from hardware performance limitations because of its complexity, large number of computations to be performed, and large number of datasets that are required for learning. To compensate for these shortcomings, an alternative called 1D-CNN has been recently developed to reduce the computational complexity by performing simple array operations instead of matrix computations performed in 2D [34,35]. 2D-CNN is an operation designed to capture the spatial relations between the neighboring pixels in an image, but time sequences of sensor data such as gyroscope or accelerometer data do not have any spatial relation. We assume that the data columns are independent and the location of the feature within the segment (fixed-length) of the overall data is not highly relevant. Therefore, we use VAE and GANomaly generation model based on 1D-CNN, which is a 1D convolution for encoding and 1D transposed convolution for decoding. The filter size is 8, the stride is 4, and the epoch value is 100. Other major hyperparameters (learning rate, lamda, and warm-up period) are automatically determined within the algorithm using BO [30].

**Figure 1:**
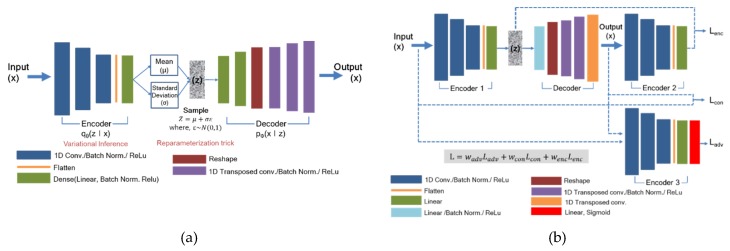
Algorithm architectures: (**a**) anomaly detection (AD) algorithm architecture using variational autoencoder (VAE) structure; (**b**) AD algorithm architecture using generative adversarial networks AD (GANomaly) structure.

The architectures of the applied VAE algorithm are constructed as shown Figure 1a and listed in Table 6 and Table 7. In VAE, we calculate an anomaly score from the reconstruction error of a given test dataset. The anomaly score function of the VAE can be derived from Equation (1) as follows:

(10) $$f_{VAE}\left(x\right)=E_{q\left(z|x\right)}\left[∥x-d_{\theta }\left(z\right){∥}_{2}^{2}\right],$$

Normal cases should have low reconstruction errors, whereas anomalies should have high reconstruction errors.

The detailed network structure and loss functions for optimization are depicted in Figure 1b, Table 8 and Table 9, respectively. During the test stage, the model uses encoder loss ($L_{enc}$) given in Equation (2) for scoring the abnormality of the given dataset. Hence, for a test sample $\hat{x}$, our anomaly score $A\left(\hat{x}\right)$ is defined with bottleneck features of the input $G_{E}\left(\hat{x}\right)$ and the encoded features of the generated output $E\left(G\left(\hat{x}\right)\right)$ as follows [24]:

(11) $$A\left(\hat{x}\right)=∥G_{E}\left(\hat{x}\right)-E\left(G\left(\hat{x}\right)\right)∥,$$

To evaluate the overall anomaly performance, we computed the anomaly score $S=\left\{s_{i}\;:A\left({\hat{x}}_{i}\right),{\hat{x}}_{i}\in \hat{D}\right\}$ for the individual test sample $\hat{x}$ within the test set $\hat{D}$. Then, we applied feature scaling to obtain the anomaly scores within the probabilistic range of [0, 1].

(12) $$s_{i}^{\prime }=\frac{s_{i}-\min\left(S\right)}{\max\left(S\right)-\min\left(S\right)},$$

### 4.3. Hyperparameter Optimization

Before training a neural network, choosing appropriate hyperparameters is crucial in obtaining a well-generalized neural network for the target task. Traditional ways to select hyperparameters include grid search and random search.

Both methods determine ranges of hyperparameter values and train the neural network by choosing hyperparameters. Although these two methods are sound ways of choosing hyperparameters, the computational complexity increases exponentially with the number of hyperparameters. To alleviate the computational complexity problem, Snoek et al. [30] proposed a BO. This BO utilizes a GP as a prior for a posterior distribution and samples a performance function whose domain is the set of hyperparameters from the posterior distribution. Finally, by choosing the most successful hyperparameters, this method can select hyperparameters without an exponentially expensive search.

In this paper, proper hyperparameters, such as learning rate and lambda for VAE, learning rate for GANomaly, and warm up period are proposed using BO. In VAE, some units of the latent variable are inactive owing to the KL-divergence term in the loss function, as shown in Equation (1), but these units remain inactive during training, preventing them from learning. This implies that the units are pruned away before they learn a useful representation. To solve this problem, Sønderby et al. propose a warm-up technique [36]. This method learns the GP based only on the reconstruction error and then gradually increases the weight of variational regularization term (KL-divergence) in loss function as the learning progresses. In this paper, not only batch normalization but also warm-up technique is applied to improve the robustness and performance of the algorithm. We use the performance indicator area under receiver operating characteristics (AUROC) as an object function to automatically find the best hyperparameters for a neural network. The related equations are described in Section 3.3.

The optimization starts with a sampling of three random hyperparameters and restricts the range of learning rate, lambda (λ) and warm up period to [10^−5^, 10^−3^], [0.2, 0.8], and [30, 500] respectively. Using the optimization method, the optimized parameters according to the two types of generation models and the performance when they are applied can be seen in Table 10.

## 5. Results and Discussion

### 5.1. Learning Models and Operation Modes

We present the AD results from the two deep-learning networks, VAE and GANomaly using data generatd by the satelite attitude control simulator LUNASIM. The reults of performance for two AD models are compared mode by mode via AUROC, as shown in Table 10. From the results, GANomaly (which utilizes GAN and AE simultaneously) gives more accurate results than VAE. This additional accuracy may arise from GANomaly’s dual minimization of both reconstruction error and JSD between input and generated samples.

Looking at the results more closely, SP mode show low AUROC values for both models (VAE and GANomaly). The SP mode is aimed at the Sun or Earth orientation in the Earth transition orbit, and includes a rotational movement of the main body for this purpose, thereby causing a large range of variation; this procedure allows a greater attitude error range than other modes. For this reason, it may be more difficult in SP mode to distinguish between normal and abnormal data. VAE is an AE variant and probability generative model that regulates latent variables to follow a multivariate normal distribution. The objective function of VAE consists of two parts: MSE for maximum likelihood estimation and KL divergence for regularization. Although the KL divergence objective allows VAE to sample more informative latent variables, the sampling process for the reparameterization can cause some noise in the reconstructed output. Therefore, when the normal data and the abnormal data are not clearly different, the VAE trained only on normal data will generate abnormal samples instead of normal ones. Conversely, in the case of GANomaly, there is no such sampling process in the middle of the neural network. Additionally, because the GANomaly utilizes AE to reconstruct input data and GAN to match a distribution of generated samples to the target distribution (distribution of normal samples), GANomaly produces less noisy data than VAE. As a result, GANomaly can discriminate abnormal samples using reconstruction error more effectively than VAE. For this reason, SP and TP modes show much better results for GANomaly than for VAE and are not suitable for applying VAE techniques. In other words, normal and abnormal data can be separated easily by VAE in modes like LAM, TSH and WOL due to the simplicity of the training data set, but such separation cannot be achieved by VAE for SP and TP, which may have some noise in the training data set.

**Figure 2:**
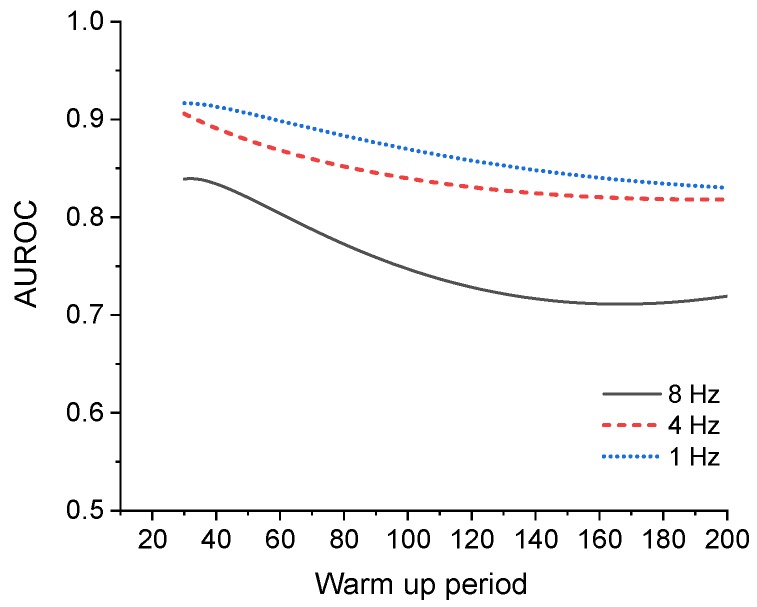
Relationship between warm up period and area under receiver operating characteristics (AUROC), (GANomaly, Sun Pointing (SP) mode).

In particular, the performance in the SP mode is lower than that in other modes. Since this is not a frequent task such as solar orientation and Earth orientation in SP mode, overfitting may occur because there are relatively many overlapping features owing to the small change in behavior. Thus, a model that fits well with the training data is created, but the performance may be degraded for the test data to be predicted. To compensate for this, learning is performed by resampling 8 Hz data to 4 Hz and 1 Hz. The results are shown in Figure 2, and the smaller the sampling value, the better the performance. The performance for the SP in Table 11 is the result of the resampled data. The raw data output from the sensor is sampled at 8 Hz, but it is confirmed that the effect of overfitting is reduced, and performance is improved as shown in Table 11 by reducing the overlapping features through down-sampling. From the results, the diagnostic results of the SP mode presented in the results (Section 5) are derived by resampling at 1 Hz.

### 5.2. Assistive Tools for AD

**Figure 3:**
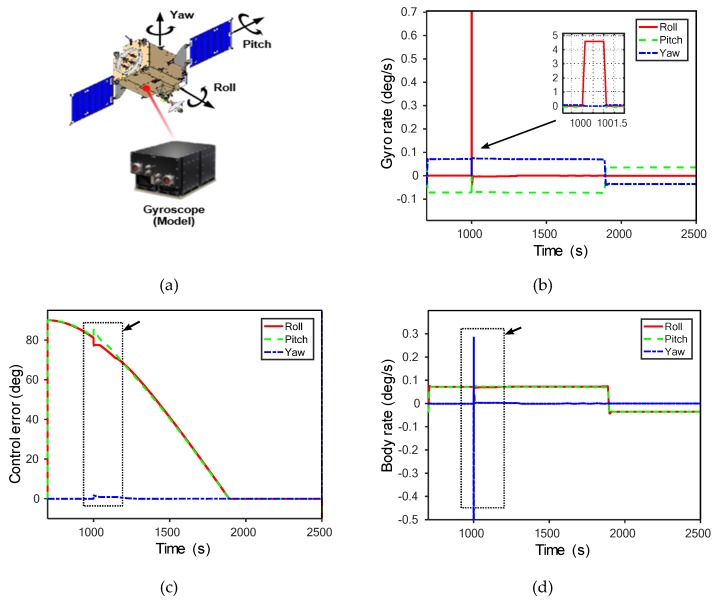
An anomaly example in LUNar Attitude and Orbit Control System SIMulator (LUNASIM): (**a**) a model example; (**b**) a fault in LUNASIM’s gyroscope model; (**c**) nonlinear changes in the modeled spacecraft control error; and (**d**) nonlinear changes in integrated angular rate.

**Figure 4:**
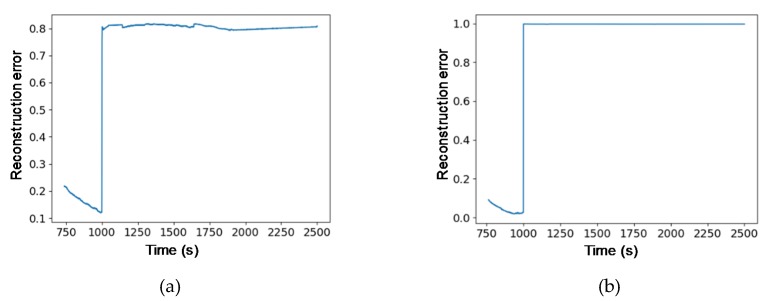
Results of AD using the deep-learning models: (**a**) the result of variational autoencoder (VAE) model; (**b**) the result of GANomaly model.

**Figure 5:**
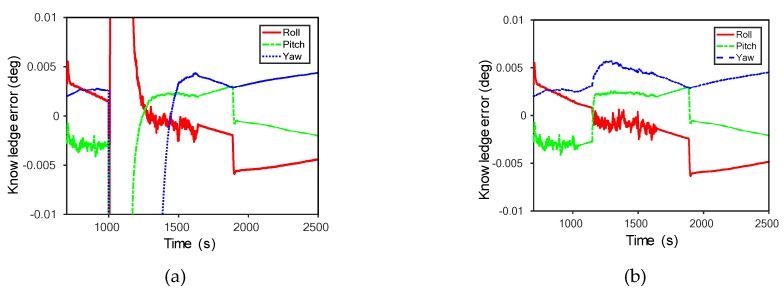
Anomaly continuing after injection point: (**a**) in case of anomaly injected; (**b**) in case of normal condition.

AD between normal and abnormal data is easier and clearer to determine using learning techniques than using monitoring of existing model output values. We look at some specific model inference examples (Figure 3a). As a simple example, we introduced a fault in LUNASIM’s gyroscope model output from 1000–1001 s (Figure 3b). Artificially increasing the gyroscope model roll rotation rate causes nonlinear changes in the modeled spacecraft control error (Figure 3c) and integrated angular rate (Figure 3d); both of these may persist for a few hundred seconds after the fault. Control error here refers to the difference between desired attitude and estimated attitude; the instantaneous deviation in the control error appears small because the fault is introduced when the control error is quite large. By applying deep-learning models to this phenomenon, distinctions between normal and abnormal can be detected, as shown in Figure 4. The sudden increase in reconstruction errors in Figure 4 after 1000 s indicates that abnormal data have occurred that are clearly different from the learned model based on normal data. Figure 5 shows that while the anomaly was injected only briefly at 1000 s, differences from normal data. Knowledge error is defined as the difference between the true attitude of the spacecraft vs the attitude estimated using data from gyroscopes and star trackers and fused using an Extended Kalman Filter; an anomalous change in gyroscope rate data, though instantaneous, may result in a persistent bias in the attitude estimate and can explain why the reconstruction error of our models does not immediately return to normal.

Monitoring for the diagnosis of spacecraft systems has been conducted with the critical point (CP) based alarm method. However, because random values are conservatively set using the expert’s experience and judgment in setting CP, frequent alarms are caused during development and operation. This makes the operator more resistant to problems, which can cause errors in troubleshooting. The deep neural network-based AD system proposed in this study can be used as a tool to improve the accuracy and reliability of the existing CP-based anomaly detection. In other words, an optimized threshold can be set based on the accumulated data.

**Figure 6:**
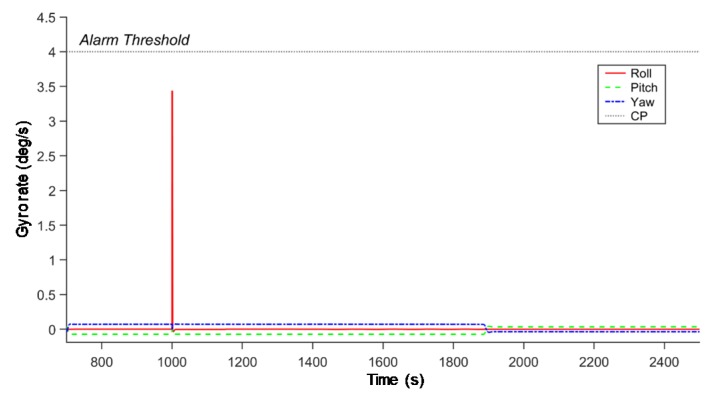
Gyro failure type of Large Angle Maneuver (LAM) mode generated under critical point (CP).

**Figure 7:**
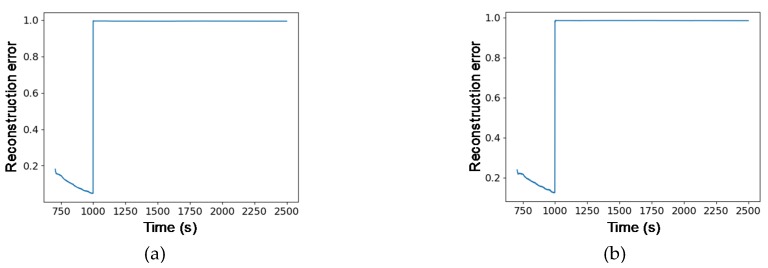
Gyro failure type detection results in LAM mode: (**a**) VAE; (**b**) GANomaly.

We randomly generate some fault signals under CP to verify the algorithm. Table 12 shows the calculation parameters and performance. In the LAM mode, a spike occurs in the Gyro rate as shown in Figure 6. From the results in Figure 7, the AD model can detect abnormalities. Therefore, in addition to the secondary role, the deep neural network-based AD model can also mechanically detect what humans cannot detect.

### 5.3. Efficient Monitoring Tool According to Purpose

AD results can be processed and monitored according to purpose. LUNASIM outputs scenario-based data, so monitoring based on operational scenarios is easy in terms of data processing. If an anomaly is detected among the modes constituting the scenario, the results of the modes are checked in detail. We present AD results from two monitoring perspective: the operation scenario and mode.

#### 5.3.1. AD in Operation Scenario

**Figure 8:**
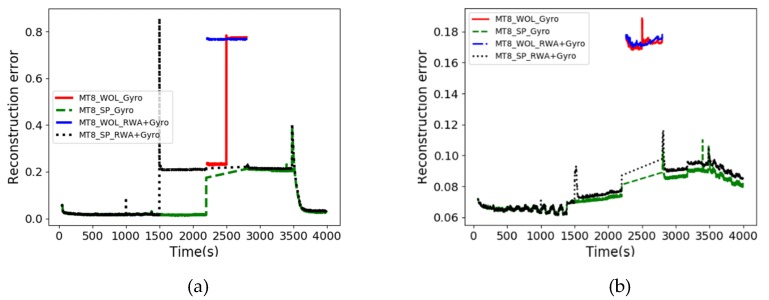
Failure types of each mode in MT8 scenario: (**a**) the result of GANomaly model; (**b**) the result of VAE model.

This method diagnoses abnormal modes for each operating scenario. Figure 8 shows the results of AD for MT8 operating scenarios in VAE- and GANomaly-based learning models. The MT8 scenario consists of SP and WOL modes. With reference to AUROCs (Table 10), both techniques in WOL mode show good detection performance, and the results of the GANomaly-based model are reliable in SP mode. In this case, it is possible to identify anomaly types in the mode as a whole in each operating scenario.

#### 5.3.2. AD in Operation Mode

**Figure 9:**
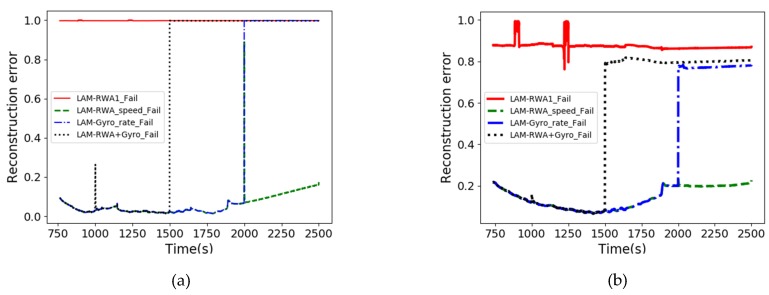
Anomaly types in LAM mode: (**a**) the result of GANomaly model; (**b**) the result of VAE model.

If an anomaly occurs during spacecraft operation, the anomaly can be detected in each operating mode. Figure 9 shows the failure types in the LAM operating mode. A failure detected with a continuous reconstruction error of 0.996 or higher indicates an RWA failure, and the peak near 1000 and 2000 s is caused by an RWA wheel speed spike. Also, a failure persisting from around 1500 to 2000 s, indicates a gyroscope rate spike. The pattern of each type of failure is distinct, so it is easy to distinguish among failures given knowledge of typical failure types.

**Figure 10:**
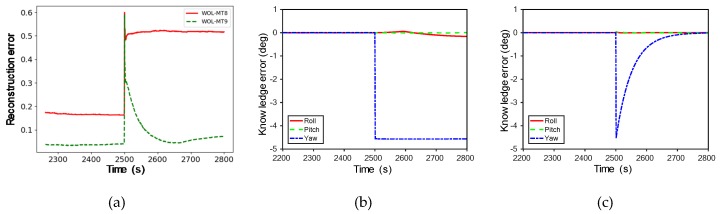
Gyro failure type of each scenario in Wheel Off Loading (WOL) mode: (**a**) the results of the MT8 and MT9 scenarios in the event of gyroscope failure in TP mode; (**b**) knowledge error in MT8 scenario; (**c**) knowledge error in MT9 scenario.

Figure 10a shows the observed results in the MT8 and MT9 scenarios in the event of gyroscope failure in TP mode. In both scenarios, failure patterns tended to be similar, followed by failures around 2500 s. Reconstruction errors are consistently high in the MT8 scenario, but not so in MT9. In this regard, the knowledge error values of gyroscopes presented in Figure 10b,c can be analyzed to determine what caused them. In the MT8 scenario, the yaw axis knowledge error was continuously high after abnormal behavior at 2500 s, and in the MT9 scenario, the value increased at the anomalous behavior but converged afterwards. As shown, even if the failure type is the same, there may be a difference in the failure occurrence pattern for each scenario. By monitoring the diagnosis result, it is possible to identify the cause of the failure in the system. If the cause is normal behavior, it is used as normal data to retrain the learning model.

#### 5.3.3. Learning Model Update

**Figure 11:**
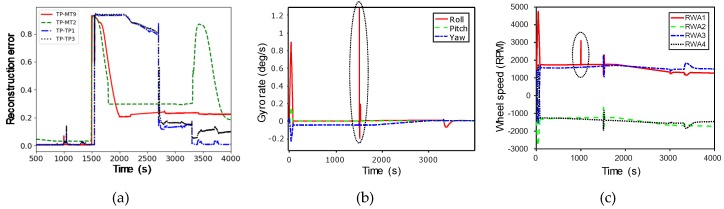
Reaction Wheels (RWA) and Gyro failure type of each scenario in Target Pointing (TP) mode: (**a**) failures in MT2, MT9, TP1, and TP3 scenario when RWA and gyro failures are mixed in TP mode; (**b**) anomalies of gyroscope rate in the MT2 scenario; (**c**) anomalies of wheel speed in the MT2 scenario.

For a single type of failure, it is possible to detect a scenario in which a failure occurs in a mode-based learning model. In other words, abnormal situations in operating scenarios can be identified in each observed mode. Figure 11a illustrates the pattern of failure in each scenario when RWA and gyro failures are mixed in TP mode.

The TP1 and TP3 scenarios show similar aspects for the same type of failure, while the MT2 and MT9 scenarios initially tend to be similar, but then present anomalies in the MT2 scenario in the vicinity of 3500 s. After checking the gyroscope rate and wheel speed of the data, as shown in Figure 11b,c, a moderately large but normal maneuver observed at 3500 s appear to be misclassified. Therefore, it is necessary to update the learning model by classifying this maneuver as normal data.

## 6. Conclusions

A machine learning-based diagnostic model is developed to perform AD on lunar orbiter AOCS. Based on the data generated by applying various scenarios to perform the exploration mission in LUNASIM, a lunar orbiter AOCS simulator, we apply semi-supervised based AD and create algorithms based on generation models such as VAE and GANomaly. Data characteristics are extracted by deep learning method such as 1D-CNN, and hyperparameters are automatically determined by applying BO with GP to improve learning performance. Anomalies are detected by measuring the generation error for newly input data. The GANomaly model performs better than VAE. Normal and abnormal data may appear similar in modes allowing a relatively large margin of error when operating in an attitude control system. In VAE, if the difference between normal and abnormal data is unclear owing to the noise caused by the reconstructed output in the sampling process for reparameterization, the VAE trained on normal data can produce abnormal samples rather than normal samples. GANomaly, however, uses reconstruction errors more effectively than VAE to distinguish abnormal samples. The reconstruction-based AD models still do not provide state-of-the-art AD performance and in this regard, we expect that a deterministic AD model may improve upon the results of this study in the future. Moreover, the accuracy and reliability of the neural networks’ abnormality detection may be improved by adding probability calibration techniques like temperature scaling and outlier exposure. In the present work, we confirm that AD models are selected based not only on the degree and kind of separation between normal and abnormal input data but also on the neural network’s processing function, process, and method. The present study also provides examples of case monitoring on a case-by-case basis, based on the spacecraft’s mission scenario and operational mode.

FD is necessary to additionally perform identification of the failure type after AD in order to be utilized more effectively in actual operation. However, this requires more data labelled for each failure type, so there is a limit to using simulation results, and actual data accumulated through long-term operation is required. After identifying the failure based on AD, the method of determining the degree of impact on the overall system performance by considering the severity of each failure and using it as a basis for decision-making can be studied as a basis for autonomous operation in the future.

## Figures and Tables

**Table 1:** Components for spacecraft attitude control system.

Units	Components	Main Output Signal
Sensors	Star sensor (STA)	Attitude (3-axis)
	Gyroscope (GRA)	Angular rate
	Coarse Sun sensor (CSSA)	Attitude (2-axis)
Actuators	Reaction wheel (RWA)	Torque

**Table 2:** List of modes.

Mode Name	Sensors(s)	Actuator(s)	Description
LAM	Star Trackers (STA) Gyros (GRA)	Reaction Wheels (RWA)	Large Angle Maneuver (LAM) to set up Del-V attitude
TP	STA, GRA	RWA	Target Pointing (TP) mode for imaging
SP	STA, GRA	RWA	Sun-Pointing (SP) mode for solar array charging and Earth communications maintenance
WOL	STA, GRA	Attitude Control Thrusters (ACT)	Wheel Off Loading (WOL) mode for dumping RWA momentum using thrusters
TSH	CSSA, GRA	ACT	Thruster-based Safe-Hold (TSH)mode just after launch vehicle separation and critical spacecraft failures

**Table 3:** List of test scenarios.

Scenario Name	Modes/Transition Times (sec)	Length (sec)
LAM1	TP (199)→ LAM (699) → SP (2500)	3664
MT1	TSH (2) → SP (1200) → TP (3999)	6000
MT2	TP (2) → SP (1799) → TP (3000)	4000
MT3	SP (2) → LAM (2100) → TP (3900)	4500
MT4	SP (2) → LAM (2100) → SP (3900)	4500
MT8	SP (2) → WOL (2199) → SP (2800)	4000
MT9	TP (2) → WOL (2199) → TP (2800)	4000
SP1	SP (2)	14,399
SP2	SP (2)	7200
TP1	TP (9)	4800
TP3	TP (9)	4800
TSH1	TSH (2)	14,399
TSH2	TSH (2)	7200
WOL1	WOL (9) → TP (999)	3000
WOL2	WOL (9) → SP (999)	3000

**Table 4:** A sample table schema.

Variable	Column Description	Units	Type
Current_time	Simulation Time	sec	Continuous
(W_body(23)-pL- > ACS_swwsc(24))*R2D	Estimated Rate Error between Model and FSW	deg	Continuous
(W_body(1)-pL- > ACS_swwsc(1))*R2D	Estimated Rate Error between Model and FSW	deg	Continuous
(W_body(2)-pL- > ACS_swwsc(2))*R2D	Estimated Rate Error between Model and FSW	deg	Continuous
AD_error(0)*R2D	Knowledge Error	deg	Continuous
AD_error(1)*R2D	Knowledge Error	deg	Continuous
AD_error(2)*R2D	Knowledge Error	deg	Continuous

**Table 5:** Summary of faults per scenario.

Scenario Name	LAM1	MT1	MT3	MT4	MT8	MT9	SP1	SP2	TP1	TP3	TSH1	TSH2	WOL1	WOL2
RWA 1,2,3,4 failure	4	-	4	4	4	4	4	4	4	4	-	-	-	-
RWA 1 wheel speed spike	4	4	4	4	4	4	4	4	4	4	-	-	4	4
Gyro rate spike	4	4	4	4	4	4	4	4	4	4	4	4	4	4
CSSA 1,4 count spike	-	1	-	-	-	-	-	-	-	-	4	4	-	-
Combined RWA1 wheel speed + Gyro rate spike	1	1	1	1	1	1	1	1	1	1	-	-	1	1
Combined CSSA spike + RWA 1 wheel speed	-	1	-	-	-	-	-	-	-	-	-	-	-	-
Combined Gyro rate + CSSA spike	-	-	-	-	-	-	-	-	-	-	1	1	-	-
Combined CSSA spike + RWA 1 wheel speed + Gyro rate spike	-	1	-	-	-	-	-	-	-	-	-	-	-	-

**Table 6:** Encoder part of VAE algorithm.

Layer	Input → Output	Operation	
**Input**	(n, 1063, 8) * → (n, 1063, 8)	Min/Max Normalization	
**Hidden**	(n, 1063, 8) → (n, 531, 4)	1D Convolution, 1D Batch Normalization, Activation Function (ReLU)	
**Hidden**	(n, 531, 4) → (n, 265, 2)	1D Convolution, 1D Batch Normalization, Activation Function (ReLU)	
**Hidden**	(n, 265, 2) → (n, 132, 1)	1D Convolution, 1D Batch Normalization, Activation Function (ReLU)	
**Flatten**	(n, 132, 1) → (n, 132)	Flatten	
**Hidden**	(n, 132) → (n, 100)	Linear, Batch Normalization, Activation Function (ReLU)	
**Output**	Mean Layer	(n, 100) → (n, 32)	Linear
	S.D. Layer	(n, 100) → (n, 32)	Linear

* (batch size, number of channels, kernel size).

**Table 7:** Decoder part of VAE algorithm.

Layer	Input → Output	Operation
**Input**	(n, 32) * → (n, 32)	Parameterization
**Hidden**	(n, 32) → (n, 100)	Linear, Batch Normalization, Activation Function (ReLU)
**Hidden**	(n, 100) → (n, 264)	Linear, Batch Normalization, Activation Function (ReLU)
**Hidden**	(n, 264) → (n, 132, 2)	Reshape
**Hidden**	(n, 132,2) → (n, 265, 4)	1D Transposed Convolution, 1D Batch Normalization, Activation Function (ReLU)
**Hidden**	(n, 265, 4) → (n, 531, 5)	1D Transposed Convolution, 1D Batch Normalization, Activation Function (ReLU)
**Output**	(n, 531, 5) → (n, 1063, 8)	1D Transposed Convolution

* (batch size, number of channels, kernel size).

**Table 8:** Encoder parts of GANomaly.

Layer	Encoder Number	Input → Output	Operation
**Input**	Ⅰ	(n, 1063, 8) * → (n, 1063, 8)	Min/Max Normalization
	Ⅱ	(n, 1063, 8)	-
	Ⅲ	(n, 1063, 8)	-
**Hidden**	Ⅰ	(n, 1063, 8) → (n, 531, 4)	1D Convolution, 1D Batch Normalization, Activation Function (ReLU)
	Ⅱ		
	Ⅲ		
**Hidden**	Ⅰ	(n, 531, 4)→ (n, 265, 2)	1D Convolution, 1D Batch Normalization, Activation Function (ReLU)
	Ⅱ		
	Ⅲ		
**Hidden**	Ⅰ	(n, 265, 2)→ (n, 132, 1)	1D Convolution, 1D Batch Normalization, Activation Function (ReLU)
	Ⅱ		
	Ⅲ		
**Flatten**	Ⅰ	(n, 132, 1) → (n, 132)	Flatten
	Ⅱ		
	Ⅲ		
**Hidden**	Ⅲ	(n, 132) → (n, 100)	Linear
**Output**	Ⅰ	(n, 132) → (n, 100)	Linear
	Ⅱ	(n, 132)→ (n, 100)	Linear
	Ⅲ	(n, 100) → (n, 1)	Linear, Sigmoid

* (batch size, number of channels, kernel size).

**Table 9:** Decoder parts of GANomaly.

Layer	Input → Output	Operation
**Input**	(n, 100) *	-
**Hidden**	(n, 100) → (n, 264)	Linear, Batch Normalization, Activation Function (ReLU)
**Hidden**	(n, 264) → (n, 132, 2)	Reshape
**Hidden**	(n, 132, 2) → (n, 265, 4)	1D Transposed Convolution, 1D Batch Normalization, Activation Function (ReLU)
**Flatten**	(n, 265, 4) → (n, 531, 5)	1D Transposed Convolution, 1D Batch Normalization, Activation Function (ReLU)
**Output**	(n, 531, 5) → (n, 1063, 8)	1D Transposed Convolution

* (batch size, number of channels, kernel size).

**Table 10:** Hyperparameters determined by Bayesian optimization.

Mode	VAE				GANomaly		
	Warm Up Period	L_r_	λ	AUROC	Warm Up Period	L_r_	AUROC
**LAM**	153.8	10^−3.022^	0.208	1.000	197.3	10^−3.305^	1.000
**SP**	300	10^−5.000^	0.200	0.686	50	10^−4.649^	0.851
**TP**	100	10^−5.000^	0.800	0.916	50	10^−5.000^	0.977
**TSH**	300	10^−4.892^	0.208	1.000	200	10^−5.000^	1.000
**WOL**	244.5	10^−0.300^	0.200	0.997	27.12	10^−5.000^	0.887

**Table 11:** Performance in the S P mode with resampled data (8 Hz, 4 Hz, 1 Hz).

SP Mode	VAE				GANomaly		
	Warm Up Period	L_r_	λ	AUROC	Warm Up Period	L_r_	AUROC
**8 Hz**	300	10^−5.000^	0.200	0.686	50	10^−4.649^	0.851
**4 Hz**	200	10^−4.999^	0.203	0.764	30	10^−2.805^	0.906
**1 Hz**	271.6	10^−5.000^	0.800	0.917	142.8	10^−1.000^	0.931

**Table 12:** Model performance for AD under CP.

Model	LAM Mode			
	Warm Up Period	L_r_	λ	AUROC
GANomaly	100.0	10^−5.0^	-	1.0
VAE	279.9	10^−5.0^	0.2	1.0
